# Adherence to perindopril/amlodipine/atorvastatin combination according to the administration strategy

**DOI:** 10.1093/ehjqcco/qcae116

**Published:** 2025-01-06

**Authors:** Gabriella Morabito, Federico Rea, Giovanni Corrao, Giuseppe Mancia

**Affiliations:** National Centre for Healthcare Research & Pharmacoepidemiology, University of Milano-Bicocca, 20126 Milan, Italy; Laboratory of Healthcare Research & Pharmacoepidemiology, Unit of Biostatistics, Epidemiology and Public Health, Department of Statistics and Quantitative Methods, University of Milano-Bicocca, 20126 Milan, Italy; National Centre for Healthcare Research & Pharmacoepidemiology, University of Milano-Bicocca, 20126 Milan, Italy; Laboratory of Healthcare Research & Pharmacoepidemiology, Unit of Biostatistics, Epidemiology and Public Health, Department of Statistics and Quantitative Methods, University of Milano-Bicocca, 20126 Milan, Italy; University of Milano-Bicocca (Emeritus Professor), Department of Statistics and Quantitative Methods, 20126 Milan, Italy; University of Milano-Bicocca (Emeritus Professor), Department of Medicine and Surgery, 20126 Milan, Italy

**Keywords:** Polypill, Adherence, Perindopril, Amlodipine, Atorvastatin, Population-based study

## Abstract

**Aims:**

To compare adherence to perindopril/amlodipine/atorvastatin combination administrated as a polypill (one pill) vs. separate tablets.

**Methods and results:**

Using the healthcare utilization database of Lombardy (Italy), 1110 patients who received the perindopril/amlodipine/atorvastatin polypill during 2019–2021 were matched with 1110 patients prescribed the same combination in separate tablets or as two antihypertensive drugs in a single tablet and the lipid-lowering drug tablet separately. Adherence to treatment was assessed over the year after the first perindopril/amlodipine/atorvastatin dispensation as the proportion of the follow-up days covered by prescription (PDC). Patients with a PDC >75% and <25% were defined as highly and poorly adherent, respectively. Adherence dynamics over time were evaluated through group-based trajectory modelling. Cardiovascular-related healthcare costs were also assessed. Log-binomial regression models were fitted to estimate the risk ratio (RR) of treatment adherence associated with the administration strategy. Among the polypill and the separate-pill combination users, 60% and 18% of patients showed high adherence, respectively; the corresponding figures for the low adherence were 5% and 37%. Compared with the separate-pill combination, the polypill increased the propensity to be highly adherent to treatment by 3.29 times (95% confidence interval: 2.88–3.75) and reduced the low adherence risk (RR = 0.13, 0.10–0.18), irrespective of sex, age, comorbidities, and co-treatment burden also throughout the entire follow-up. The polypill was also associated with lower costs (€676 vs. €1068, *P* = 0.003).

**Conclusion:**

In a real-life setting, the polypill improved treatment adherence and reduced healthcare costs compared to the corresponding separate-pill combination. These findings support current guidelines in favour of the polypill.

## Graphical Abstract

**Graphical Abstract:**
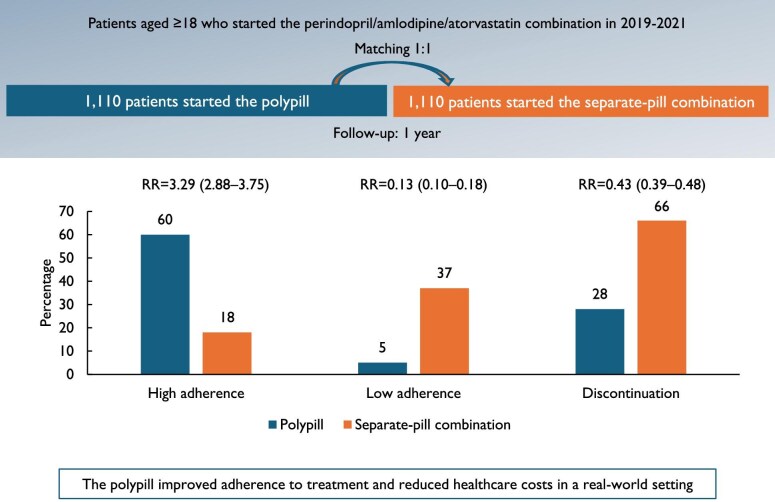

Key learning points **What is already known**

- Several clinical trials showed that the administration of antihypertensive and lipid-lowering drugs in a single pill improves adherence to treatment.

**What this study adds**

- This study investigated in a real-world setting the beneficial effect of the perindopril/amlodipine/atorvastatin polypill vs the corresponding separate-pill combination on treatment adherence.

## Introduction

High blood pressure (BP) and elevated low-density lipoprotein cholesterol (LDL-C) are two of the modifiable risk factors for cardiovascular (CV) disease to which a large fraction of premature CV death can be attributed.^1^ Although many treatment options are available, BP and LDC-C control rates are still low, mainly due to poor medication adherence,^2,3^ whose relationship with increased CV morbidity and mortality is widely known.^4,5^ Since the coexistence of elevated BP and LDL-C is frequent and has a multiplicative effect on CV risk,^6^ removing barriers against adherence to either pharmacological treatment is a key goal to be achieved. In this context, (i) reduction of the daily number of tablets to be assumed improves adherence to several drug treatments, including antihypertensive and lipid-lowering treatments,^7^ (ii) this improvement has been found to be associated with a reduction of CV events and mortality,^4,5^ and (iii) the combination in a single tablet of two BP-lowering agents and a statin, referred to as a polypill, has been found to both enhance adherence and lower CV outcomes in clinical trials on either primary and secondary CV prevention patients, those in CV prevention frequently with the addition of aspirin.^8–14^ However, drug adherence as evaluated in clinical trials may not reflect the actual medication adherence in clinical practice and real-world evidence on this issue is still scant, i.e. limited to a few studies focusing on polypills comprising aspirin.^15,16^

The aim of the present study was to compare adherence to a combination of two antihypertensive agents and a statin administrated as a polypill vs. separate tablets (two or three pills) in a real-world setting of Italy. Since the only polypill of this kind available in Italy at the time of the study consisted of perindopril, amlodipine, and atorvastatin, the investigation was restricted to these drugs. Comparison of the two treatment strategies was extended to health care costs for CV services.

## Methods

### Setting

Data were retrieved from the Healthcare Utilization Databases of Lombardy, a region of Italy that accounts for almost 16% (approximately 10 million) of its population, which has equal access to the healthcare services provided by the National Health Service (NHS). In each Italian region, an automated system of databases collects information on the provided health services free of charge, including demographic and administrative data of NHS beneficiaries, outpatient drug dispensing (classified according to the Anatomical Therapeutic Chemical—ATC—classification system), hospital discharge records (with diagnoses and procedures coded according to the International Classification of Diseases, 9th Revision, Clinical Modification—ICD-9-CM—classification system), and specialist visits and diagnostic examinations. A single, individual identification code allows us to link these databases and trace the healthcare pathway of NHS beneficiaries. To preserve privacy, each identification code was automatically deidentified, the inverse process being only allowed to the Regional Health Authority upon request from judicial authorities. Further details on healthcare utilization databases of the Lombardy Region employed in pharmacoepidemiological studies are available in previous publications.^17,18^

According to Italian law, studies based entirely on registry data are exempt from patient’s informed consent and do not require approval from an ethics review board (General Authorization for the Processing of Personal Data for Scientific Research Purposes Issued by the Italian Privacy Authority on 10 August 2018; https://www.garanteprivacy.it/web/guest/home/docweb/-/docweb-display/docweb/9124510).

### Cohort selection and follow-up

The target population included all Lombardy residents aged 18 years or older. Among them, those who received a combination of perindopril, amlodipine, and atorvastatin either as a polypill or in separate tablets between 2019 and 2021 were identified. Polypill of perindopril, amlodipine and atorvastatin included the following concentrations of each component: 5 mg/5 mg/10 mg; 10 mg/10 mg/20 mg; 10 mg/5 mg/20 mg; 5 mg/5 mg/20 mg; 10 mg/10 mg/40 mg. Separate-pill administration included a dispensation of perindopril and amlodipine as a single-pill combination (SPC) plus a dispensation of atorvastatin (i.e. two pills) or the three drugs given separately. The SPC of amlodipine/atorvastatin and perindopril/atorvastatin were not available in the Italian market. The date of the first concomitant dispensation of the perindopril/amlodipine/atorvastatin combination during 2019–2021 was defined as the *index date*. Patients were excluded if they (i) were not beneficiaries of the NHS for at least 5 years before the index date, (ii) had less than 1 year of follow-up (because of death or change of residence from Lombardy to other regions/states), and (iii) did not renew the drug prescriptions. The cohort members were followed from the index date for 365 days afterwards.

### Adherence to and discontinuation of perindopril/amlodipine/atorvastatin

For each cohort member, all perindopril, amlodipine, and atorvastatin dispensations during the follow-up were identified. The period covered by a prescription was calculated by dividing the total amount of the drug dispensed for the defined daily dose (perindopril and amlodipine) and the number of tablets in the dispensed canister (atorvastatin). For overlapping prescriptions, the patient was assumed to have taken all the drug(s) contained in the former prescription before starting the latter one. Adherence to drug therapy was assessed by the ratio between the number of days in which the three drugs were available and the days of follow-up, a measure referred to as ‘proportion of days covered’ (PDC) by prescriptions.^19^ Two analyses were performed to compare adherence between groups. First, the association of drug administration strategy with the odds of being highly adherent to treatment (PDC >75%) was estimated. Second, by means of group-based trajectory modelling,^20^ the dynamics of drug adherence over time were investigated and patients were classified into relatively homogeneous subgroups based on their trajectory.

Secondary aims were to compare the two groups for the odds of being poorly adherent (PDC <25%) and of discontinuing treatment. Starting from the index drug dispensation, consecutively refilled prescriptions were considered uninterrupted if the time span between the end of the first one and the beginning of the following one was less than 90 days. If the between-prescription timespan was longer, treatment discontinuation was assumed.^19^

### Covariates

Baseline characteristics included sex, age, previous use of antihypertensive and statin drugs, potency of atorvastatin at the index date, use of other drugs (e.g. antidiabetic drugs), and previous hospitalization for selected conditions. In addition, the number of co-medications dispensed in the year prior to the index date was assessed and categorized as 0–3, 4–8, and ≥9. Finally, the clinical profile was assessed by the multisource comorbidity score, a prognostic score that has been shown to predict all-cause death of Italian people more accurately than other widely used scores.^21^ Three categories of clinical profile were considered: good (0≤ score ≤3), intermediate (4≤ score ≤7), and poor (score ≥8).

### Healthcare costs

Healthcare costs were calculated by the amount that the Regional Health Authority reimbursed to health providers. Costs included hospitalization and emergency room visits for CV diseases, antihypertensive and lipid-lowering drugs, and outpatient services for CV care (specialist visits, laboratory examinations, imaging, etc.). Healthcare costs were assessed for the year after the index date.

### Data analysis

Cohort members were classified based on the treatment strategy (i.e. polypill vs. separate-pill combination) at the index date according to the intention-to-treat approach.

The chi-square test, or its version for the trend, (categorical covariates) and the *t*-test (continuous covariates) were used to test for differences between groups.

Because patients prescribed the polypill and the separate-pill combination could be different for several characteristics, the high-dimensional propensity score (hdPS) matching design was adopted.^22^ Compared with manual identification of confounders, this data-driven approach is able to identify pre-exposure variables that favour information on important unmeasured confounders. The propensity score of being prescribed the polypill was obtained through a logistic regression model that included the aforementioned covariates plus the *k* most predictive covariates selected by the algorithm among all causes of hospitalization experienced by, and all drugs dispensed to cohort members over the 5-year period prior to the index date. To optimize hdPS with respect to the number *k* of empirically identified covariates to be included in the model, a Super Learner approach was adopted.^23^ Six hdPS candidate models with 20, 40, 80, 120, 160, and 200 empirically identified covariates, respectively, were fitted and incorporated into the Super Learner to obtain a weighted prediction (i.e. the final propensity score). For each patient prescribed the polypill, all potential comparators were identified among patients prescribed the separate-pill combination with an index date that fell within ±30 days of the index date of the corresponding polypill patient. Groups were then matched 1:1 based on their propensity score, using a nearest neighbour matching algorithm without replacement.

Log-binomial regression models were fitted to estimate the risk ratios (RR), and their 95% confidence interval (CI), of treatment adherence and discontinuation in relation to drug strategy, using the separate-pill combination as reference.

The selection of the number of trajectory groups was based on model fit indices, i.e. Bayesian information criteria and Akaike information criterion.

A multinomial logistic regression model was fitted to estimate the association between patients’ characteristics and adherence trajectories.

Analyses were then repeated, stratifying patients for sex, age, number of comedications, and clinical status.

### Sensitivity analyses

To verify the robustness of our findings, three further analyses were performed. First, because patients on the separate-pill combination could assume drugs at a lower daily dosage than that used for the calculation of adherence, we increased the coverage of each prescription in patients of this group until the observed association between the administration strategy and treatment adherence was nullified. This approach allows detecting the magnitude of the between-group difference in daily dosage required to fully explain the exposure effect on adherence. Second, adherence to and discontinuation of the two drug therapies (i.e. antihypertensive drugs or atorvastatin) were decomposed and considered separately. Third, patients on the separate-pill combination were stratified based on the antihypertensive drug strategy (i.e. SPC or two pills).

The Statistical Analysis System Software (version 9.4; SAS Institute, Cary, North Carolina, USA) was used for the analyses. For all hypotheses tested, two-tailed *P* values <0.05 were significant.

## Results

### Patients

Among the 1441 patients prescribed the perindopril/amlodipine/atorvastatin polypill during 2019–2021, 1125 individuals met the inclusion criteria. Of these, 1110 patients were matched to 1110 out of 12 723 eligible subjects prescribed a separate-pill combination of the drugs of interest. Characteristics of the two groups before matching and propensity score distributions before and after matching are reported in Supplementary material online, *Table S1* and *Figure S1*.

As shown in *Table 1*, groups had superimposable baseline characteristics after matching. About three out of five patients were men, the mean age was over 66 years, almost 90% and 60% of patients were already treated with antihypertensive and statin drugs before the index date, and one out of five patients had a poor clinical status.

**Table 1:** Characteristics of patients prescribed the polypill and of the matched patients prescribed the corresponding separate-pill combination

	Patients on polypill (*N* = 1110)	Patients on separate-pill combination (*N* = 1110)	*P*-value
Men	692 (62.3)	702 (63.2)	0.661
Age (years): mean [SD]	66.9 [11.0]	66.4 [11.0]	0.262
High potency atorvastatin at the index date	842 (75.9)	868 (78.2)	0.190
Previous antihypertensive drug strategy			0.226
No antihypertensive drugs	122 (11.0)	111 (10.0)	
Monotherapy	162 (14.6)	154 (13.9)	
Dual combination	350 (31.5)	396 (35.7)	
Three or more drugs	476 (42.9)	449 (40.5)	
Previous statins use	639 (57.6)	647 (58.3)	0.731
Other drugs			
Antidiabetic drugs	303 (27.3)	287 (25.9)	0.442
Anticoagulant agents	86 (7.8)	78 (7.0)	0.516
Antiplatelet agents	373 (33.6)	405 (36.5)	0.155
Antidepressant drugs	171 (15.4)	184 (16.6)	0.452
Number of co-treatments			0.535
0–3	250 (22.5)	266 (24.0)	
4–8	435 (39.2)	411 (39.2)	
≥9	425 (38.3)	433 (38.3)	
Previous hospitalizations			
Stroke	46 (4.1)	45 (4.1)	0.915
Heart failure	21 (1.9)	27 (2.4)	0.381
Myocardial infarction	41 (3.7)	49 (4.4)	0.389
Diabetes	56 (5.1)	55 (5.0)	0.922
Cancer	71 (6.4)	74 (6.7)	0.797
Clinical profile^a^			0.227
Good	656 (59.1)	672 (60.5)	
Intermediate	253 (22.8)	221 (19.9)	
Poor	201 (18.1)	217 (19.6)	

a Three categories were considered for the clinical profile according to the multisource comorbidity score: good (0≤ score ≤3), intermediate (4≤ score ≤7), and poor (score ≥8).

### Adherence to and discontinuation of perindopril/amlodipine/atorvastatin

**Figure 1:**
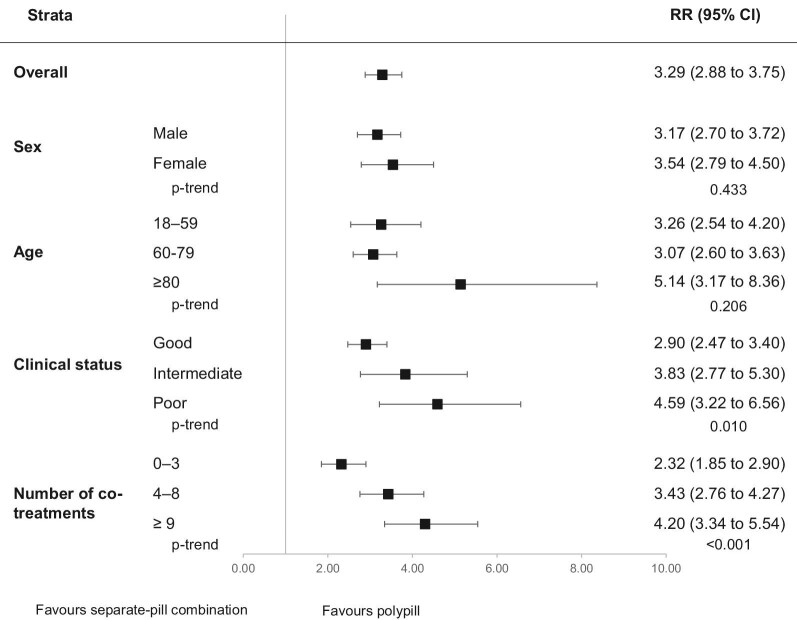
Risk ratios (RR), and 95% confidence intervals (CI), estimating the association between high adherence to treatment (PDC >75%) and the polypill vs. the separate-pill combination.

High adherence to treatment (PDC >75%) was observed in 60% of patients prescribed the polypill and in 18% of those prescribed the three-drug combination given separately. As shown in *Figure 1*, compared to those prescribed the separate-pill combination, patients on the polypill had a greater chance of being highly adherent to treatment (RR = 3.29, 95% CI: 2.88–3.76, *P* < 0.001), irrespective of age, sex, clinical status, and number of co-medications. The benefit was greater among patients with a more impaired clinical status (*P* = 0.010) and a higher medication burden (*P* < 0.001).

**Figure 2:**
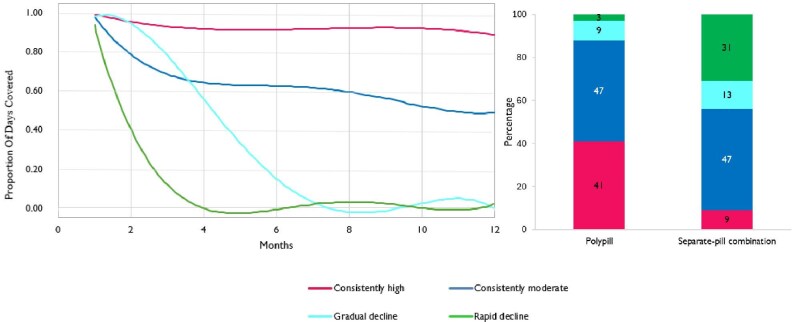
Adherence trajectory patterns and proportion of patients in each trajectory according to drug strategy (polypill vs. separate-pill combination).

Four different adherence trajectories were identified (*Figure 2*). These patterns can be described as follows: (i) consistently high, (ii) consistently moderate, (iii) gradual decline, and (iv) rapid decline. There was evidence that, compared to those prescribed the separate-pill combination, patients on the polypill were more likely to have a consistently high adherence (41% vs. 9%) and less likely to have a rapid decline (3% vs. 31%) (*P* < 0.001). Baseline characteristics of patients according to their trajectory pattern are reported in Supplementary material online, *Table S2*, while Supplementary material online, *Figure S2* shows the association estimates between patients’ characteristics and adherence trajectories. Women, older patients, patients with previous admission for heart failure, previous use of antiplatelets, a higher medication burden, and a more impaired clinical status were more likely to have a gradual or rapid decline in adherence. Conversely, those with previous use of antihypertensive drugs and statins, as well as patients hospitalized for diabetes showed a lower propensity to gradually or rapidly reduce adherence.

**Figure 3:**
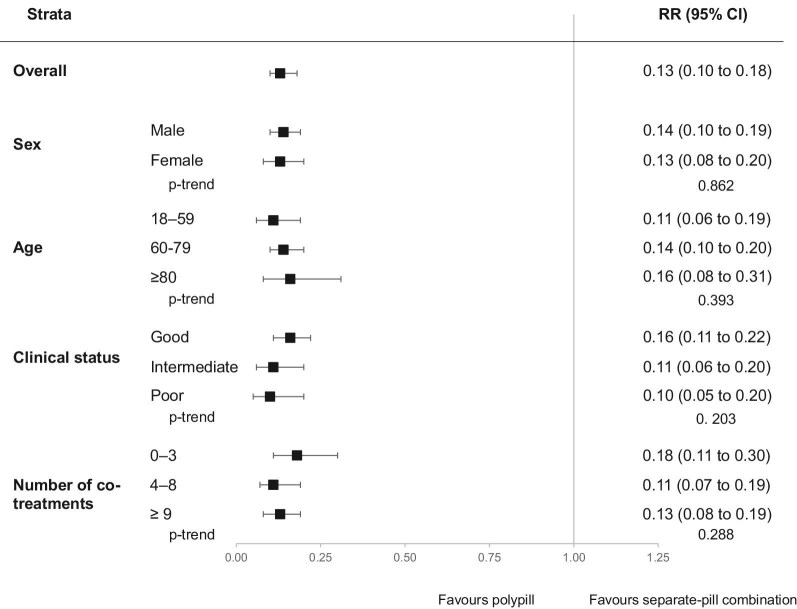
Risk ratios (RR), and 95% confidence intervals (CI), estimating the association between low adherence to treatment (PDC <25%) and the polypill vs. the separate-pill combination.

Low adherence to therapy (PDC <25%) was observed in 5% and 37% of patients prescribed the polypill and the separate-pill combination, respectively. As shown in *Figure 3*, compared to patients using separate pills, those on the polypill had a lower risk of low adherence to drug therapy (RR = 0.13, 95% CI: 0.10–0.18, *P* < 0.001). This was the case for all strata of age, sex, clinical status, and number of co-medications.

**Figure 4:**
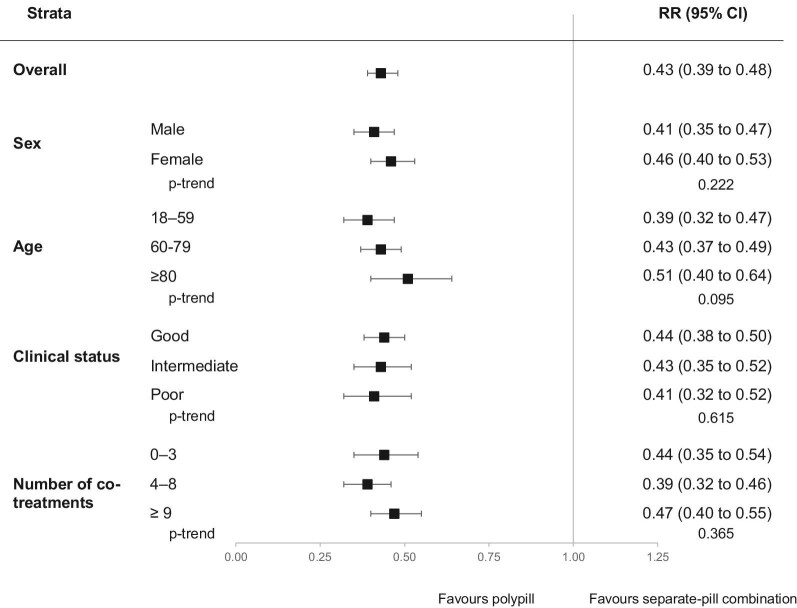
Risk ratios (RR), and 95% confidence intervals (CI), estimating the association between treatment discontinuation and the polypill vs. the separate-pill combination.

Discontinuation of drug treatment was observed in 28% of patients prescribed the polypill and in 66% of those prescribed the three-drug combination given separately. As shown in *Figure 4*, compared to those prescribed the separate-pill combination, patients on the polypill had a lower risk of treatment discontinuation (RR = 0.43, 95% CI: 0.39–0.48, *P* < 0.001), irrespective of age, sex, clinical status, and number of co-medications.

### Healthcare costs

Compared to patients on the separate-pill combination, the mean healthcare cost was significantly lower among those on polypill (€676 vs. €1068, *P* = 0.003). This was the case for CV drugs (i.e. antihypertensive and statin drugs) (€241 vs. €295), for hospital admission (€373 vs. €700) and emergency room visits for CV events (€6 vs. €14), and for outpatient services for CV care (€56 vs. €60).

### Sensitivity analyses

Supplementary material online, *Figure S3* shows that patients on the separate-pill combination should have reduced the daily dosage at least three times to nullify the observed between-group difference in treatment adherence.

The analysis of adherence to and discontinuation of specific drug therapies showed that the improvement associated with the polypill was similar between antihypertensive drugs and atorvastatin (Supplementary material online, *Table S3*).

As shown in Supplementary material online, *Table S4*, the polypill was associated with a greater chance of being adherent to treatment and a lower risk of discontinuation compared to both patients prescribed the perindopril/amlodipine in a SPC plus the atorvastatin tablet separately and those prescribed all drugs (atorvastatin plus the two antihypertensive drugs) in separate pills.

## Discussion

The present real-world investigation provides several novel findings. Compared with separate-pill administration, the polypill had a beneficial effect on the adherence and persistence to either antihypertensive and statin treatment, regardless of sex, age, clinical status, and concomitant pill burden. These advantages were by no means quantitatively marginal because the polypill (i) increased the chance to be highly adherent to treatment by more than three times, with the improvement being more pronounced in patients with a more impaired clinical status and a higher amount of co-medication, (ii) reduced the risk of being poorly adherent to therapy by 87%, and (iii) reduced the risk of discontinuing treatment by 57%. Furthermore, the risk of low adherence over time (i.e. the ‘rapid decline’ of adherence trajectory) was lower among patients prescribed the polypill. This improved adherence to treatment might have a major clinical impact because poor medication adherence and discontinuation have been shown to increase the risk of hospitalization and mortality in several studies.^3,4,24,25^

Several other findings deserve attention. First, our study confirmed that adherence to CV medications is low in clinical practice,^3,4^ and that this also applied to BP-lowering agents and statins used in combination. Second, in previous investigations based on the Lombardy population, we have reported that adherence to and discontinuation of CV drug therapies is affected by a number of patient's demographic and clinical characteristics, and even by the density of the population where the patient lives.^4,26–28^ In this study we add that another factor to take into account can be the adherence trajectories, which can show marked between-patient differences, although the polypill administration effectively improved adherence in all trajectory subgroups. This provides a background that can help the practicing physician in his/her attempt to judge the risk of non-adherence in a given patient, with the important limitation that measuring adherence at the individual patient level remains extremely difficult. Third, the polypill was used by only 8% of patients prescribed the perindopril/amlodipine/atorvastatin combination; hence, given the benefits associated with this administration strategy, its wider use should be encouraged. Fourth, compared with the separate-pill combination, the polypill was associated with lower cost for all CV-related health services, with the largest reduction being observed in hospitalization spending. This result suggests that the polypill may be a cost-saving strategy, with advantages both for patients and for the NHS.^29^ Fifth, the polypill conferred an equal improvement in adherence to antihypertensive drugs and atorvastatin, suggesting that the factor responsible for this beneficial effect is probably treatment simplification.

This study has strengths and limitations. Among the former, our investigation was conducted on a very large and unselected population because Italy's NHS includes all citizens. Additionally, our databases provide highly accurate data as health providers are required to report services in detail to obtain reimbursement, with legal consequences for incorrect reports.^30^ Measuring adherence with such databases also allowed to avoid patients’ behavioural changes induced by the awareness of being monitored.^31^ Finally, the results were confirmed by several sensitivity analyses.

On the other hand, some limitations should be mentioned. One, our data include drug dispensations within the NHS reimbursement system and not those provided at the private practice level.^17^ However, given their free of charge availability, prescriptions of CV drugs outside NHS are known to be only 6% of the total amount,^32^ making misclassification of drug exposure in the overall population unlikely. Two, since the prescribed daily doses are not recorded in our database, we calculated drug coverage based on the defined daily dose (perindopril and amlodipine) and on the number of tablets in the dispensed canister (atorvastatin). It is possible that patients on the separate-pill administration used lower doses of the drugs, leading to an overestimation of the polypill's adherence benefit. However, our sensitivity analysis showed that only a sizeable and unrealistic between-group difference in the daily drug dosage would have nullified this effect, reinforcing the robustness of the observed benefit. Three, during the observation period, 0.4% and 1.4% of patients switched from the separate-pill combination to the polypill and vice versa, respectively. Though not accounted for in our intention-to-treat analysis, these low rates are unlikely to have significantly influenced the results. Four, owing to privacy rules, hospital records were not available for scrutiny, which means that CV diagnoses could not be checked.^33^ Also, due to the limited amount of clinical data, our database cannot provide information on factors such as differences in achieved BP, serum LDL-C and side effects between the polypill and no-polypill treatment strategies. However, it is likely that, compared with the separate-pill administration, the polypill led to better BP and LDL-C control because of the well-established association between adherence to CV drugs and reduction of BP and LDL-C levels.^2,3,34,35^ Lastly, because of the limited statistical power, we could not explore in this study whether, compared to the no-polypill treatment strategy, the polypill was associated with a reduced risk of CV outcomes. Nor could we address whether different adherence trajectories were accompanied by different levels of CV risk. However, as far as the former issue is concerned, improved adherence to BP or lipid-lowering treatment has been repeatedly a protective effect on CV outcomes and mortality.^3,4^

In conclusion, in our real-world study, the polypill substantially improved treatment adherence compared with the corresponding separate-pill administration, irrespective of age, sex, clinical status, and concurrent pill load. In addition, the polypill reduced CV-related healthcare costs. These findings strongly support current guidelines in favour of treatment regimen simplification and advocate for the widespread use of the polypill when appropriate.

## Supplementary Material

qcae116_Supplemental_File

## Notes

**Conflict of interest.** Giovanni Corrao received research support from the European Community (EC), the Italian Agency of Drug (AIFA), and the Italian Ministry of Education, University and Research (MIUR). He took part to a variety of projects that were funded by pharmaceutical companies (i.e. Novartis, GSK, Roche, AMGEN, BMS, and Servier). He also received honoraria as member of Advisory Board from Roche. Giuseppe Mancia received honoraria for participation as speaker/chairman in national/international meetings from Bayer, Boehringer Ingelheim, CVRx, Daiichi Sankyo, Ferrer, Medtronic, Menarini Int., Merck, Novartis, Recordati, and Servier. Federico Rea received research support from Servier. Gabriella Morabito declares that she has no competing interests.

## Data availability

The data that support the findings of this study are available from Lombardy Region but restrictions apply to the availability of these data, which were used under license for the current study, and so are not publicly available. Data are however available upon reasonable request and with permission of Lombardy Region. Requests to access these datasets should be directed to DG Welfare Lombardy Region, Epidemiology Observatory Organisational Unit (olivia_leoni@regione.lombardia.it).
