# Supplementary material for: Adherence to perindopril/amlodipine/atorvastatin combination according to the administration strategy
Source: Eur Heart J Qual Care Clin Outcomes. 2025 Jan 6;11(8):1301–9. doi: 10.1093/ehjqcco/qcae116 (PMC12714380; doi:10.1093/ehjqcco/qcae116)

**Adherence to perindopril/amlodipine/atorvastatin combination according to the administration strategy**

Gabriella MORABITO ^1,2^, Federico REA ^1,2^, Giovanni CORRAO ^3^, Giuseppe MANCIA ^3^

^1^ National Centre for Healthcare Research & Pharmacoepidemiology, at the University of Milano-Bicocca Milan, Italy

^2^ Laboratory of Healthcare Research & Pharmacoepidemiology, Unit of Biostatistics, Epidemiology and Public Health, Department of Statistics and Quantitative Methods, University of Milano-Bicocca, Milan, Italy

^3^ University of Milano-Bicocca (Emeritus Professor), Milan, Italy

**SUPPLEMENTARY MATERIAL**

**Summary**

[**Supplementary Table S1**. Characteristics of patients prescribed the polypill and of the patients prescribed the corresponding separate-pill combination before matching. 3](#_Toc179289754)

[**Supplementary Table S2.** Patients’ characteristics according to adherence trajectories. 4](#_Toc179289755)

[**Supplementary Table S3.** Risk ratios (RR), and 95% confidence intervals (CI), estimating the association between drug treatment strategy (polypill vs. separate-pill combination) and high adherence to and discontinuation of the two drug treatments considered separately (i.e., perindopril/amlodipine or atorvastatin). 6](#_Toc179289756)

[**Supplementary Table S4.** Risk ratios (RR), and 95% confidence intervals (CI), estimating the association between drug treatment strategy (polypill vs. separate-pill combination) and high adherence to and discontinuation of drug treatment, stratifying patients on separate-pill combination according to their antihypertensive drug strategy (i.e., single-pill combination – SPC – or two pills). 7](#_Toc179289757)

[**Supplementary Figure S1.** Propensity score distributions, according to the treatment group, before and after matching. 8](#_Toc179289758)

[**Supplementary Figure S2.** Odds ratios, and their 95% confidence intervals, estimating the association between adherence trajectories and patients’ characteristics. 9](#_Toc179289759)

[**Supplementary Figure S3.** Risk ratios (RR), and 95% confidence intervals, estimating the association between high adherence to treatment (PDC>75%) and treatment strategy by varying the ratio of daily dose dispensed between patients under the polypill and the separate-pill combination. 10](#_Toc179289760)

# **Supplementary Table S1**. Characteristics of patients prescribed the polypill and of the patients prescribed the corresponding separate-pill combination before matching.

|  | **Patients on polypill**  **(N=1,125)** | **Patients on separate-pill combination**  **(N=12,723)** | **p-value** |
| --- | --- | --- | --- |
| Men | 704 (62.6) | 7,755 (61.0) | 0.284 |
| Age (years): mean [SD] | 66.9 [11.1] | 70.7 [10.8] | <0.001 |
| High potency atorvastatin at the index date | 855 (76.0) | 8,939 (70.3) | <0.001 |
| Previous antihypertensive drug strategy |  |  | <0.001 |
| No antihypertensive drugs | 123 (10.9) | 297 (2.3) |  |
| Monotherapy | 168 (14.9) | 426 (3.4) |  |
| Dual combination | 350 (31.1) | 4,856 (38.2) |  |
| Three or more drugs | 484 (42.9) | 7,144 (56.2) |  |
| Previous statins use | 646 (57.4) | 11,083 (87.1) | <0.001 |
| Other drugs |  |  |  |
| Antidiabetic drugs | 309 (27.5) | 3,724 (29.3) | 0.202 |
| Anticoagulant agents | 86 (7.6) | 1,311 (10.3) | 0.005 |
| Antiplatelet agents | 376 (33.4) | 6,117 (48.1) | <0.001 |
| Antidepressant drugs | 173 (15.4) | 2,348 (18.4) | 0.010 |
| Number of co-treatments |  |  | <0.001 |
| 0–3 | 252 (22.4) | 1,948 (15.3) |  |
| 4–8 | 439 (39.0) | 4,873 (38.3) |  |
| ≥9 | 434 (38.6) | 5,902 (46.4) |  |
| Previous hospitalizations |  |  |  |
| Stroke | 47 (4.2) | 877 (6.9) | <0.001 |
| Heart failure | 22 (2.0) | 295 (2.3) | 0.435 |
| Myocardial infarction | 41 (3.6) | 667 (5.2) | 0.020 |
| Diabetes | 59 (5.2) | 727 (5.7) | 0.514 |
| Cancer | 74 (6.6) | 991 (7.8) | 0.144 |
| Clinical profile ^§^ |  |  | <0.001 |
| Good | 660 (58.7) | 6,346 (49.9) |  |
| Intermediate | 257 (22.8) | 3,375 (26.5) |  |
| Poor | 208 (18.5) | 3,002 (23.6) |  |

^§^ Three categories were considered for the clinical profile according to the Multisource Comorbidity Score: good (0 ≤ score ≤ 3), intermediate (4 ≤ score ≤ 7), and poor (score ≥ 8).

# **Supplementary Table S2.** Patients’ characteristics according to adherence trajectories.

|  | **Consistently high (N=563)** | **Consistently moderate (N=1,037)** | **Gradual decline (N=248)** | **Rapid decline (N=372)** | **p-value** |
| --- | --- | --- | --- | --- | --- |
| Men | 371 (65.9) | 667 (64.3) | 139 (56.0) | 217 (58.3) | 0.010 |
| Age (years) |  |  |  |  | 0.086 |
| 18-59 | 163 (29.0) | 281 (27.1) | 53 (21.4) | 113 (30.4) |  |
| 60-79 | 334 (59.3) | 606 (58.4) | 150 (60.5) | 206 (55.4) |  |
| ≥ 80 | 66 (11.7) | 150 (14.5) | 45 (18.1) | 53 (14.2) |  |
| High potency atorvastatin at the index date | 438 (77.8) | 793 (76.5) | 195 (78.6) | 284 (76.3) | 0.843 |
| Previous antihypertensive drug strategy |  |  |  |  | 0.082 |
| No antihypertensive drugs | 47 (8.3) | 112 (10.8) | 30 (12.1) | 44 (11.8) |  |
| Monotherapy | 75 (13.3) | 140 (13.5) | 35 (14.1) | 66 (17.7) |  |
| Dual combination | 203 (36.1) | 342 (33.0) | 71 (28.6) | 130 (34.9) |  |
| Three or more drugs | 238 (42.3) | 443 (42.7) | 112 (45.2) | 132 (35.5) |  |
| Previous statin use | 344 (61.1) | 606 (58.4) | 152 (61.3) | 184 (49.5) | 0.002 |
| Other drugs |  |  |  |  |  |
| Antidiabetic drugs | 147 (26.1) | 286 (27.6) | 61 (24.6) | 96 (25.8) | 0.751 |
| Anticoagulant agents | 33 (5.9) | 81 (7.8) | 19 (7.7) | 31 (8.3) | 0.434 |
| Antiplatelet agents | 178 (31.6) | 372 (35.9) | 99 (39.9) | 129 (34.7) | 0.120 |
| Antidepressant | 81 (14.4) | 172 (16.6) | 38 (15.3) | 64 (17.2) | 0.606 |
| Number of co-treatments |  |  |  |  | 0.005 |
| 0–3 | 133 (23.6) | 249 (24.0) | 41 (16.5) | 93 (25.0) |  |
| 4–8 | 233 (41.4) | 401 (38.7) | 90 (36.3) | 122 (32.8) |  |
| ≥9 | 197 (35.0) | 387 (37.3) | 117 (47.2) | 157 (42.2) |  |
| Previous hospitalizations |  |  |  |  |  |
| Stroke | 33 (5.9) | 30 (2.9) | 14 (5.6) | 14 (3.8) | 0.019 |
| Heart failure | 7 (1.2) | 19 (1.8) | 12 (4.8) | 10 (2.7) | 0.008 |
| Myocardial infarction | 22 (3.9) | 34 (3.3) | 19 (7.7) | 15 (4.0) | 0.019 |
| Diabetes | 35 (6.2) | 46 (4.4) | 11 (4.4) | 19 (5.1) | 0.453 |
| Cancer | 43 (7.6) | 62 (6.0) | 15 (6.0) | 25 (6.7) | 0.623 |
| Clinical profile ^§^ |  |  |  |  | 0.337 |
| Good | 343 (60.9) | 628 (60.6) | 140 (56.5) | 217 (58.3) |  |
| Intermediate | 127 (22.6) | 219 (21.1) | 56 (22.6) | 72 (19.4) |  |
| Poor | 93 (16.5) | 190 (18.3) | 52 (21.0) | 83 (22.3) |  |

^§^ Three categories were considered for the clinical profile according to the Multisource Comorbidity Score: good (0 ≤ score ≤ 3), intermediate (4 ≤ score ≤ 7), and poor (score ≥ 8).

# **Supplementary Table S3.** Risk ratios (RR), and 95% confidence intervals (CI), estimating the association between drug treatment strategy (polypill vs. separate-pill combination) and high adherence to and discontinuation of the two drug treatments considered separately (i.e., perindopril/amlodipine or atorvastatin).

| **Outcome** | **RR (95% CI)** | **Patients on polypill (%)** | **Patients on separate-pill combination (%)** |
| --- | --- | --- | --- |
| Adherence to atorvastatin | 1.51 (1.40–1.63) | 66.6 | 43.8 |
| Adherence to perindopril/indapamide | 1.42 (1.31–1.54) | 61.5 | 43.1 |
| Discontinuation of atorvastatin | 0.50 (0.44–0.57) | 21.1 | 41.9 |
| Discontinuation of perindopril/amlodipine | 0.61 (0.54–0.68) | 26.5 | 43.8 |

# **Supplementary Table S4.** Risk ratios (RR), and 95% confidence intervals (CI), estimating the association between drug treatment strategy (polypill vs. separate-pill combination) and high adherence to and discontinuation of drug treatment, stratifying patients on separate-pill combination according to their antihypertensive drug strategy (i.e., single-pill combination – SPC – or two pills).

| **Outcome** | **Antihypertensive drug strategy** | **RR** | **95% CI** |
| --- | --- | --- | --- |
| High adherence | SPC | 3.17 | 2.76–3.65 |
| High adherence | Two pills | 4.17 | 2.85–6.11 |
| Discontinuation | SPC | 0.44 | 0.39–0.48 |
| Discontinuation | Two pills | 0.40 | 0.35–0.46 |

# **Supplementary Figure S1.** Propensity score distributions, according to the treatment group, before and after matching.

**
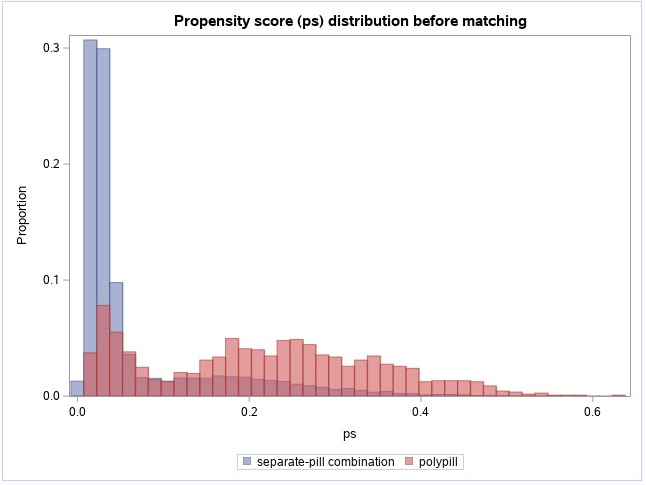
**


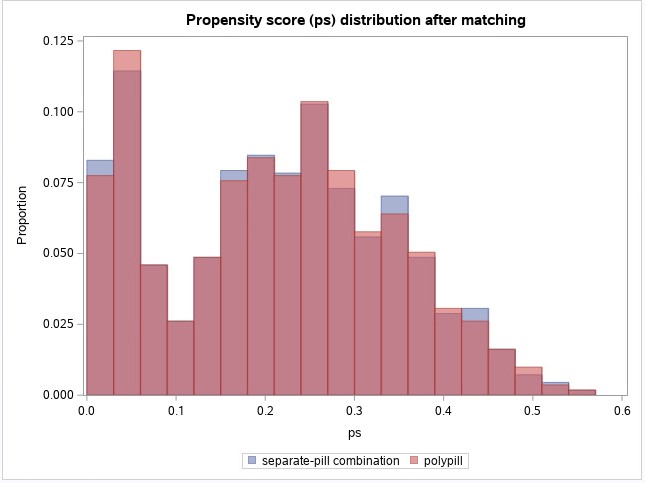


# **
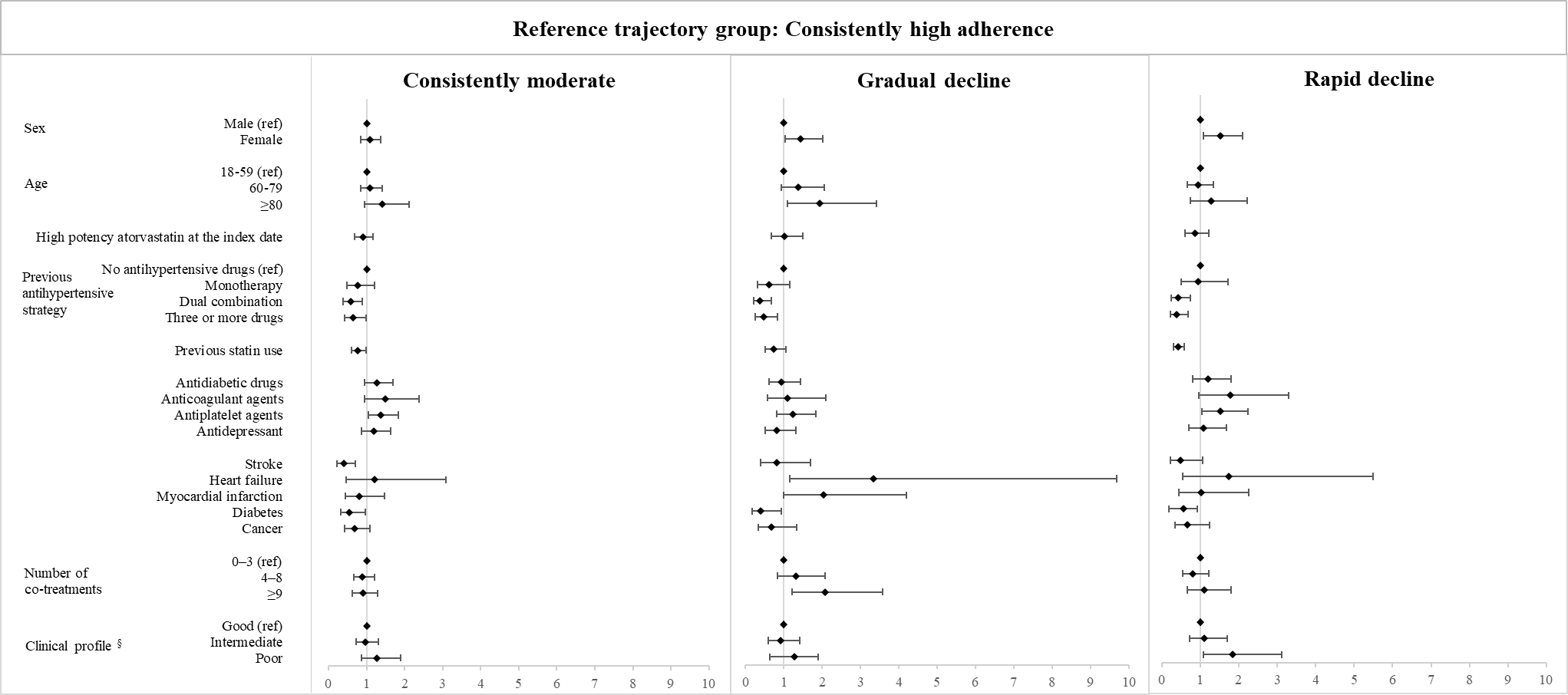
Supplementary Figure S2.** Odds ratios, and their 95% confidence intervals, estimating the association between adherence trajectories and patients’ characteristics.

^§^ Three categories were considered for the clinical profile according to the Multisource Comorbidity Score: good (0 ≤ score ≤ 3), intermediate (4 ≤ score ≤ 7), and poor (score ≥ 8).

# **Supplementary Figure S3.** Risk ratios (RR), and 95% confidence intervals, estimating the association between high adherence to treatment (PDC>75%) and treatment strategy by varying the ratio of daily dose dispensed between patients under the polypill and the separate-pill combination.


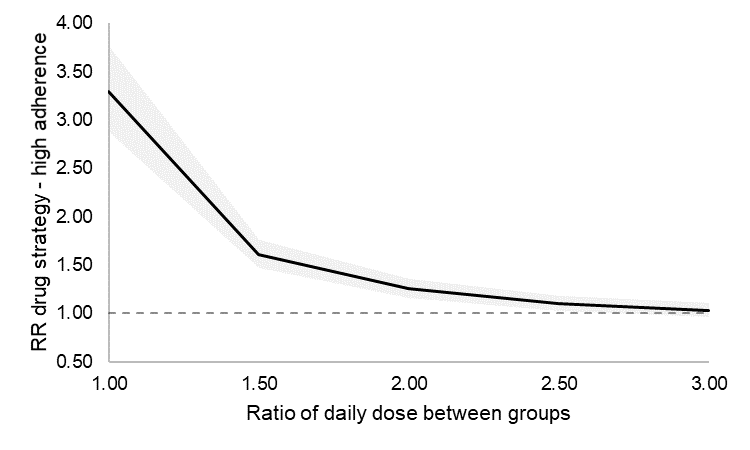

Supplement: qcae116_Supplemental_File [file qcae116_supplemental_file.docx]
